# Alternating dose-dense chemotherapy in patients with high volume disseminated non-seminomatous germ cell tumours

**DOI:** 10.1038/sj.bjc.6600272

**Published:** 2002-05-03

**Authors:** K Fizazi, D M Prow, K-A Do, X Wang, L Finn, J Kim, D Daliani, C N Papandreou, S-M Tu, R E Millikan, L C Pagliaro, C J Logothetis, R J Amato

**Affiliations:** Department of Genitourinary Medical Oncology and Department of Biostatistics, The University of Texas M. D. Anderson Cancer Center, Houston, Texas, USA

**Keywords:** chemotherapy, dose-dense chemotherapy, germ-cell tumours, International Germ-Cell Cancer Consensus Group, non-seminomatous germ cell-tumours

## Abstract

Only about half of patients with a poor-prognosis non-seminomatous germ-cell tumours can achieve a cure. The aim of this phase II study was to assess the efficacy and toxicity of a dose-dense alternating chemotherapy regimen in this subset of patients. High volume non-seminomatous germ-cell tumours was defined as follows: at least two sites of non pulmonary metastases, an extragonadal primary tumour, a serum human chorionic gonadotropin level higher than 10 000 mIU ml^−1^, or a alpha-foetoprotein level higher than 2000 mIU ml^−1^. Patients who fulfilled these criteria were treated with the so-called BOP-CISCA-POMB-ACE regimen (bleomycin, vincristine, and cisplatin; cisplatin, cyclophosphamide, and doxorubicin; cisplatin, vincristine, methotrexate, and bleomycin; etoposide, dactinomycin, and cyclophosphamide) plus granulocyte colony-stimulating factor. A total of 58 patients were enrolled. Patients were retrospectively classified according to the International Germ-Cell Cancer Consensus Group classification; 38 patients (66%) had poor-prognosis disease and 19 patients (33%) had intermediate-prognosis. Patients received a median of 2.5 courses (range 0.25 to five courses) of the BOP-CISCA-POMB-ACE regimen. Forty-two patients (72.4%) had a complete response to therapy. With a median follow-up time of 31 months, the 3-year progression-free survival rate was 71% (95% confidence interval, 60 to 84%) and the 3-year overall survival rate was 73% (95% confidence interval: 62 to 86%). The 3-year PFS rates were 83% (95% confidence interval: 68 to 100%) in the intermediate-prognosis group and 65% (95% confidence interval: 51 to 82%) in the poor-prognosis group. Early side effects included mainly grade 4 haematologic toxicity (neutropaenia in 79% of patients, thrombocytopaenia in 69%, anaemia in 22%), grade 4 stomatitis (19%), and four early deaths (7% of patients), at least partially related to toxicity. The dose-dense BOP-CISCA-POMB-ACE regimen is highly active in patients with non-seminomatous germ-cell tumours classified as intermediate-prognosis or poor-prognosis according to the International Germ-Cell Cancer Consensus Group. Because outcomes with this regimen compare favourably with outcome after standard therapy, dose-dense chemotherapy should be further investigated in this subset of patients.

*British Journal of Cancer* (2002) **86**, 1555–1560. DOI: 10.1038/sj/bjc/6600272 www.bjcancer.com

© 2002 Cancer Research UK

## 

The use of cisplatin-based chemotherapy and surgery in the first-line treatment of non-seminomatous germ-cell tumours (NSGCT) has resulted in cure rates of approximately 80% during the past two decades (Bosl and Motzer, 1997; Culine *et al*, 1996). Improved treatment has led to the need to stratify patients by prognosis. Although many groups have identified clinical and biological features of NSGCT with prognostic relevance, there are considerable differences between the prognostic classifications that have emerged from these analyses. These differences make it difficult to compare results of clinical trials. In 1997, the International Germ-Cell Cancer Consensus Group (IGCCCG) published a consensus prognostic index for NSGCT (International Germ-Cell Cancer Consensus Classification, 1997). This index stratifies patients into good-, intermediate-, and poor-prognosis subgroups on the basis of three criteria: the primary tumour site, the levels of serum tumour markers, and whether extra-pulmonary visceral metastases are present. This index has been validated by the IGCCCG and by other groups (Bower *et al*, 1997; Hinton *et al*, 2000). Patients allocated to the good-prognosis group have a high probability of cure, but patients in the intermediate-prognosis and poor-prognosis groups have 3-year survival rates of only 81% and 50%, respectively.

Since the recognition in the 1980s of a group of patients with advanced NSGCT with a poor prognosis, a number of attempts have been made to improve the cure rate in this subgroup. In 1987, investigators from Indiana University published a subgroup analysis of a randomised trial that compared four cycles of cisplatin, bleomycin, and etoposide (BEP) *vs* cisplatin, vinblastine, and bleomycin regimen (PVB). The analysis showed that BEP produces better outcomes in patients with poor-prognosis NSGCT, and the BEP became the standard in this subgroup (Williams *et al*, 1987). Subsequent studies that have attempted to identify a regimen superior to BEP in this setting have focused on increasing the peak dose intensity (Ozols *et al*, 1988; Nichols *et al*, 1991; Fizazi, 2002), using high-dose chemotherapy with autologous haematopoietic stem cell support (Baume *et al*, 1990; Chevreau *et al*, 1993), integrating ifosfamide into first-line chemotherapy (Nichols *et al*, 1998; de Wit *et al*, 1998), and developing dose-dense alternating regimens (Kaye *et al*, 1998; Fizazi and Zelek, 2000). So far, randomised trials have failed to identify a regimen with efficacy superior to that of BEP.

It has been suggested that increased dose intensity may lead to improved results in germ-cell tumours (Samson *et al*, 1984; Bokemeyer *et al*, 1999). However, on the basis of the results from two randomised studies (Nichols *et al*, 1991; Chevreau *et al*, 1993) and of our own trial comparing two doses of the CISCA/VB regimen (Fizazi *et al*, 2002), we believe that increasing the peak doses is not likely to improve cure. Indeed, this strategy results in a significant delay between courses of chemotherapy and limits the number of potentially active agents that may be used to circumvent resistance to therapy. Another way to increase dose intensity is to increase ‘dose density’ – that is, to decrease the interval over which a specified dose is given. This strategy has proven feasible and has now been used worldwide in the treatment of a great variety of neoplasms (Fizazi and Zelek, 2000). Taking advantage of the facts that several drugs effective against NSGCT – including bleomycin, vincristine, cisplatin, and methotrexate – are not haematotoxic or are moderately haematotoxic and that hematopoietic growth factors are available, we designed a dose-dense multidrug regimen for high volume NSGCT, called BOP-CISCA-POMB-ACE. This regimen is a hybrid of three regimens previously shown to be individually effective against NSGCT: BOP (bleomycin, vincristine, and cisplatin), which was developed at the University of Colorado (Wettlaufer *et al*, 1984), CISCA (cisplatin, cyclophosphamide, and doxorubicin) which was developed at The University of Texas M.D. Anderson Cancer Center (Fizazi *et al*, 2002; Logothetis *et al*, 1985, 1986), and POMB/ACE (cisplatin, vincristine, methotrexate, and bleomycin, followed by etoposide, dactinomycin, and cyclophosphamide), which has been used since 1979 at the Charing Cross Hospital, London (Bower *et al*, 1997). Here we report the results of a phase II study of this regimen.

## PATIENTS AND METHODS

### Eligibility

This was a prospective phase II trial. Signed informed consent forms approved by the M. D. Anderson Cancer Center's Institutional Review Board were obtained from all patients before enrolment. All patients had histologically confirmed NSGCT, evidence of dissemination, and a high disease volume according to the modified M. D. Anderson classification (Logothetis *et al*, 1986). High-volume NSGCT was defined by the presence of at least one of the following criteria: at least two sites of non-pulmonary visceral metastases, an extragonadal primary tumour, a serum human chorionic gonadotropin (hCG) level higher than 10 000 mIU ml^−1^, or an alpha-foetoprotein (AFP) higher than 2000 mIU ml^−1^. Additional inclusion criteria included a white blood cell count of at least 3000 mm^3^, a platelets count of at least 100 000 mm^3^, a creatinine clearance level of greater than 40 ml min^−1^, a bilirubin level less than 1.5 times the upper limit of normal, and alanine transaminase level of no more than four times the upper limit of normal. Excluded from this study were patients infected by the human immunodeficiency virus (they are the subject of a separate report (Fizazi *et al*, 2001)) and patients who had previously undergone chemotherapy. There were no exclusion criteria based on performance status or life expectancy.

### Baseline assessment

Baseline assessment included a review of the histologic tumour type and serum tumour marker assays for hCG, AFP, and lactate dehydrogenase (LDH). Radiographic studies at baseline included chest radiography, and computed tomography (CT scan) of the pelvis, abdomen and brain. A computed tomography of the chest was performed in cases of suspicious thoracic lesions. A bone scan was also performed when clinically indicated. A gallium scan of the lung and a forced vital capacity measurement (FVC) were also performed. Other baseline studies included a dental evaluation, an audiogram, an electrocardiogram, an echocardiogram, a complete blood count with platelet count, and an assessment of electrolytes, testosterone level, and renal and liver function. Additional studies were obtained as warranted by the patient's clinical presentation. Sonography of the contralateral testis was performed to detect contralateral tumours.

### Evaluation and supportive care during chemotherapy

A complete blood count with platelet count was obtained three times a week. A physical examination and assessment of electrolytes, predicted creatinine clearance, and serum tumour markers were performed before each course of chemotherapy. A FVC was performed before each course that included bleomycin. If a drop in the FVC of 10% or more below the baseline level was observed and confirmed, bleomycin was discontinued.

Patients were instructed to go to the hospital for a complete blood count if they developed a fever. Broad-spectrum antibiotics were started in the case of febrile neutropenia. Platelet transfusion and red cell transfusion were also used in the case of severe thrombocytopenia (platelets level less than 10 000 mm^3^) and severe anaemia (haemoglobin level less than 7 g dl^−1^). Intensive mouth washes and acyclovir were used in the case of severe stomatitis.

### Treatment

Induction chemotherapy consisted in the BOP-CISCA-POMB-ACE regimen, the details of which are outlined in Table 1

**Table 1:**
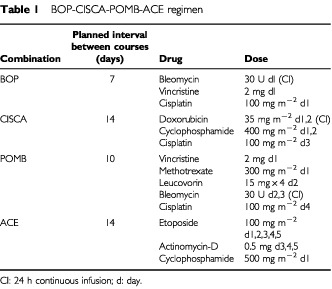
BOP-CISCA-POMB-ACE regimen

. Bleomycin was given as a 24-h continuous infusion. Cisplatin was delivered over a period of 2 h with simultaneous forced mannitol diuresis as previously described (Hayes *et al*, 1977). Vincristine, etoposide and dactinomycin were administered as an intravenous bolus. Doxorubicin was given as a 48-h continuous infusion. Cyclophosphamide was given as a 1-h intravenous infusion. Methotrexate was given as a 12-h continuous infusion and followed by leucovorin rescue. Leucovorin was given 24-h after the start of the methotrexate infusion at a dose of 15 mg as an intravenous bolus every 12 h for four courses. Granulocyte colony-stimulating factor (G-CSF) was administered subcutaneously at a dose of 5 μg kg day^−1^ beginning the day after completion of chemotherapy. G-CSF was continued for a minimum of 5 days and until the absolute granulocyte count was higher than 1000 mm^3^. Chemotherapy was started 24 h after the discontinuation of G-CSF and according to the schedule summarised in Table 1 provided that the absolute granulocyte count was higher than 1000 mm^3^, the platelet count was higher than 50 000 mm^3^ (without a decreasing trend), and any other grade 3 or 4 toxic effects had resolved. The total number of chemotherapy courses was individualised on the basis of tumour response. Patients received one cycle of the BOP-CISCA-POMB-ACE regimen beyond the one in which complete remission was achieved or in which a radiographically stable mass and normal levels of serum biomarkers were established. In the case of a radiographically stable mass, exploratory surgery was performed to remove residual masses. Patients who were found at surgery to have viable carcinoma received two courses of salvage chemotherapy, typically cisplatin, ifosfamide, and etoposide. Patients with brain metastases and remaining masses after chemotherapy were treated with either neurosurgery or radiotherapy; the choice of treatment was based upon the extent of the disease and the patient's underlying medical condition.

### Assessment of response

Response was assessed after every complete cycle of BOP-CISCA-POMB-ACE. Initially abnormal radiologic exams and serum tumour markers were obtained. Patients in whom tumour markers normalised and for whom there was no clinical or radiologic evidence of disease were classified as having had a complete response to chemotherapy (cCR). Patients with normal findings on tumour marker studies and in whom completely resected residual masses contained only necrosis or mature teratoma were classified as having had a pathologic complete response (pCR). Patients with normal tumour markers and in whom completely resected masses contained viable cancer were classified as having had a surgical complete response (sCR). Patients in whom tumour marker levels failed to normalise, although a plateau was reached were classified as having had an incomplete response (IR) and were proposed surgery or salvage chemotherapy. All other responses were considered incomplete responses. Progressive disease (PD) was defined as rising tumour marker levels confirmed at least twice or the appearance of new lesions, except when pathologic evidence of a growing teratoma syndrome (Logothetis *et al*, 1982; André *et al*, 2000) was provided. Before response assessment, patients with tumour markers on plateau were subjected to either surgical resection of residual masses or follow-up of tumour markers. Patients who normalised tumour markers after surgery were classified as either pCR or sCR according to whether viable cancer was found at pathological examination. Patients who were not submitted to surgery were classified as progression in case of tumour marker raise after the plateau.

### Statistical analysis

This phase II trial was conducted in two stages using the method of Simon (Simon 1989) to determine whether the BOP-CISCA-POMB-ACE regimen was likely to be of sufficient efficacy to be worthy of further study. The main end-point of the trial was the complete response rate. The following two hypotheses were considered: H_0_; *P*⩽0.30, a complete response rate of no interest, *vs* H_1_; *P*⩾0.50, a complete response rate that would be of considerable interest in further studies. The study plan was as follows: 22 patients would be entered in the first stage of the study, and 24 would be entered in the second stage, for a total of 46 patients. If the complete response rate was 32% (7 out of 22) or less at the end of the first stage, the study would be stopped. Otherwise, accrual would continue. If the final complete response rate was 37% (17 out of 46) or less, the regimen would not be studied further. If the complete response rate was higher, the regimen would be recommended for further study. If the true complete response rate was 30%, the expected sample size for the trial would be 30, and the probability of early termination would be 67%. These calculations assumed type I and type II error rates of 10% (i.e., there is ⩾90% probability that the regimen would be recommended for further study if the true efficacy rate is ⩾50% and <10% probability if the true efficacy rate is ⩽30%). After the IGCCCG criteria were published in 1997 (3), it was decided to expand the study to reduce the confidence interval of the subgroup of patients belonging to the poor-prognosis group according to the IGCCCG.

Survival curves were generated by the method of Kaplan and Meier. Overall survival (OS) was calculated from the date of enrolment in the trial to the last date of follow-up. Progression-free survival (PFS) was calculated from the date of inclusion into the trial to the date of progression or relapse or to the date of last follow-up. All analyses were performed using S-PLUS statistical software.

## RESULTS

### Therapeutic outcome

Fifty-eight patients with high volume disseminated NSGCT were enrolled in this trial between October 1993 and April 1998. All patients were eligible and were included in the analysis. There were 57 males and one female. Patient characteristics are summarised in Table 2

**Table 2:**
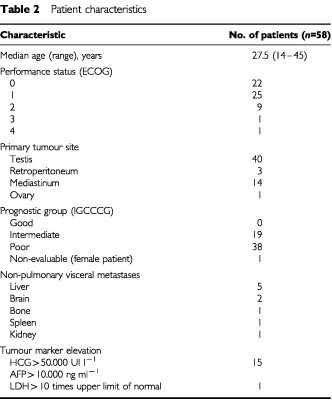
Patient characteristics

. Patients were retrospectively classified according to the IGCCCG classification; 38 patients (66%) had poor-prognosis disease and 19 patients (33%) had intermediate-prognosis disease. Seventeen patients had an extragonadal primary tumour site.

Patients received a median of 2.5 courses of the BOP-CISCA-POMB-ACE regimen (range 0.25 to 5 courses). Response to therapy is summarised in Table 3

**Table 3:**
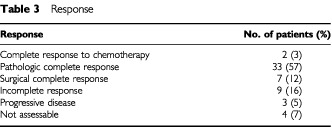
Response

. Response was assessed in an intend to treat basis. Overall, 42 patients (72.4%) had a complete response to therapy, including seven patients who had viable non-teratomatous malignant cells in their resected residual masses. Nine patients had an incomplete response including three who had a partial response with serum tumour marker normalisation. Residual masses were not resected in these three patients because of anatomic location of brain metastases (one patient), insufficient FVC to allow extensive thoracic surgery (one patient), and patient refusal (one patient). Four patients (6.8%) were not assessable for response because of early death.

**Figure 1:**
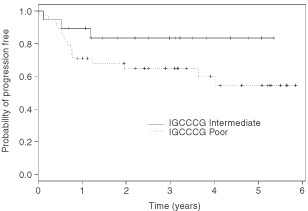
Progression-free survival according to IGCCCCG groups.

After a median follow-up time of 31 months (range, 0.3 to 71 months), the 3-year PFS rate for the entire population was 71% (95% confidence interval [CI], 60 to 84%) and the 3-year OS rate was 73% (95% CI, 62 to 86%). The 3-year PFS rates were 83% (95% CI, 68 to 100%) in the intermediate-prognosis group and 65% (95% CI, 51 to 82%) in the poor-prognosis group. The 3-year OS rates were 83% (95% CI, 67 to 100%) in the intermediate-prognosis group and 67% (95% CI, 53 to 84%) in the poor-prognosis group. Survival curves according to IGCCCG groups are shown in Figure 1

. The 3-year OS rate in patients with primary mediastinal NSGCT was 64% (95% CI, 44 to 95%).

### Toxicity and causes of death

Overall, the BOP-CISCA-POMB-ACE regimen was associated with a high incidence of toxic effects. Table 4

**Table 4:**
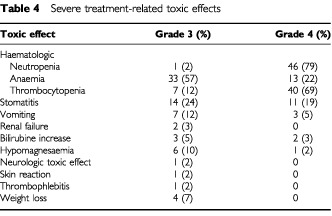
Severe treatment-related toxic effects

summarises the distribution of grade 3 and 4 toxic effects. Haematologic and gastrointestinal side effects (stomatitis and vomiting) were among the most frequent side effects, even though prophylactic measures were used. Peripheral neuropathy also occurred often (grade 1: 17%, grade 2: 19%, grade 3: 2%). Four patients died of toxicity-related deaths. One patient died of a subarachnoid haemorrhage secondary to thrombocytopaenia. Another patient, with a history of polycystic bilateral kidney disease and a high-volume NSGCT with bulky retroperitoneal nodes compressing the uretera and causing destruction of the lumbar vertebra, developed an acute renal insufficiency and died of associated complications. Two patients died of sepsis. Another patient developed acute leukaemia (refractory anaemia with excess blasts in transformation) 14 months after a complete remission of NSGCT was documented. At the time of this report, this patient is alive 2 years after treatment of his hematologic malignancy. One patient died of pneumonia 3 years after chemotherapy was completed and a complete remission of a primary mediastinal NSGCT had been documented.

## DISCUSSION

Patients with intermediate-prognosis and poor-prognosis NSGCT according to the IGCCCG classification have 3-year OS rates of only 81 and 50%, respectively (International Germ-Cell Cancer Consensus Classification, 1997). The 3-year OS rates in our prospective phase II study of the dose-dense BOP-CISCA-POMB-ACE regimen (83 and 67% in the intermediate- and in the poor-prognosis group, respectively) compare favourably with the IGCCCG rates and also with those achieved with the BEP regimen (Hinton *et al*, 2000; Kaye *et al*, 1998) although a direct comparison is not possible since ours was not a randomised study.

In recent years, data have accumulated suggesting that a high cure rate can be achieved in patients with intermediate- and poor-prognosis NSGCT when dose-dense regimens are used (Bower *et al*, 1997; Kaye *et al*, 1998; Fizazi and Zelek, 2000; Lewis *et al*, 1991; Horwich *et al*, 1994, 1997; Harstrick *et al*, 1991; Germa Lluch *et al*, 1992, 1999). However, among the studies that have addressed this issue, only a few (Horwich *et al*, 1997) were designed as ‘true’ formal phase II studies like the present study, and only one was a randomised trial (Kaye 1998). One of the oldest dose-dense schedules, the POMB/ACE regimen, consists of a combination of seven drugs, recycled alternatively at 2-week intervals. In patients with intermediate-prognosis and poor-prognosis NSGCT according to the IGCCCG criteria, 3-year survival with this regimen are 88 and 75%, respectively (Bower *et al*, 1997). Another accelerated induction regimen, the so-called C-BOP-BEP regimen, has been developed at the Royal Marsden Hospital in London (Horwich *et al*, 1994, 1997). This weekly regimen is a combination of cisplatin, bleomycin, vincristine, etoposide, and carboplatin. Results are very promising: in 41 patients with poor-prognosis according to the IGCCCG criteria, the 3-year OS rate was 91%. Surprisingly, results in intermediate-risk patients were more disappointing, as the 3-year OS rate was only 68% (Horwich *et al*, 1997). This regimen is now being evaluated in a phase II trial by the European Organization for Research and Treatment of Cancer (EORTC). The German Cooperative Group for Testicular Cancer has reported its own experience with a rapidly alternating combination. The 3-year OS rate in 48 patients with poor-risk NSGCT according to this group's classification was 76% (Harstrick *et al*, 1991). Similarly, investigators of the Spanish Germ-Cell Cancer Group have developed an intensive alternating multidrug schedule, the so-called BOMP/EPI regimen that produced a 64% 2-year OS rate in 38 patients with poor-prognosis disease according to the IGCCCG criteria (Germa Lluch *et al*, 1992, 1999). Finally the Medical Research Council and the EORTC have reported the results of a phase III trial that compared a rapidly recycled intensive regimen (BOP/VIP-B) to the standard 4 BEP in 380 patients with poor-prognosis NSGCT (Kaye *et al*, 1998). Unfortunately, the BOP/VIP-B regimen, which was initially reported to be highly efficient in 91 patients treated in an uncontrolled study (Lewis *et al*, 1991) did not afford any survival benefit in this randomised study. Possible explanations for these negative results are a higher number of toxicity-related deaths (12 *vs* 4) and a higher proportion (10% *vs* 5%) of primary mediastinal NSGCT, notorious for their poor outcome (Fizazi *et al*, 1998), in the intensive arm.

Although prophylactic G-CSF was used in our study, the toxicity of the BOP-CISCA-POMB-ACE regimen was high, although manageable in most cases. Four early deaths occurred that were, at least in part, related to treatment toxicity. One patient developed a secondary leukaemia, a phenomenon that has been extensively described in germ-cell tumour survivors (Bokemeyer and Schmoll, 1995). Relatively high rates of toxicity-related death have previously been reported in patients treated with dose-dense regimens (Bower *et al*, 1997; Kaye *et al*, 1998; Lewis *et al*, 1991; Horwich *et al*, 1994, 1997; Harstrick *et al*, 1991; Germa Lluch *et al*, 1992, 1999). For example, 6.5% and 7% patients died of toxicity after they had received the BOP/VIP-B and the C-BOP-BEP regimens, respectively (Kaye *et al*, 1998; Horwitch *et al*, 1997). However, it is noteworthy that the toxicity-related death rate of patients with poor-prognosis NSGCT treated with standard regimens is not negligible. Indeed, although the toxicity-related death rate associated with the standard BEP regimen is typically low (around 1%) in good-prognosis patients (Cvitkovic, 1998) this rate ranges from 2% to 6.4% in poor-prognosis patients (Nichols *et al*, 1991; Kaye *et al*, 1998; Droz *et al*, 2001). There are at least two possible ways to reduce the toxicity-related death rate associated with dose-dense regimens in poor-prognosis patients in the future. First, these regimens may be optimised with the goals of reducing the incidence of profound grade 4 thrombocytopenia and neutropenia and of limiting the total dose of bleomycin, especially in patients with multiple lung metastases (for example those with the ‘choriocarcinoma syndrome’). Second, since about half of patients with a poor-prognosis according to the IGCCCG are cured with the BEP regimen, we need to improve the selection of patients for potentially more active regimens so that patients who can be cured with BEP are spared the added toxicity of these regimens. Some authors have suggested that subgroups with different survival rates can be identified in the poor-prognosis IGCCCG group (Kollmannsberger *et al*, 2000). Investigators from Memorial Sloan-Kettering Cancer Center have suggested that tumour marker half-life correlates with survival (Toner *et al*, 1990), although investigators from the Royal Marsden Hospital did not confirm these findings (Stevens *et al*, 1995). Whether a slow decrease in tumour marker levels is a predictor of poor outcome in patients with poor-prognosis or intermediate-prognosis NSGCT according to the IGCCCG was very recently suggested (Mazumdar *et al*, 2001). This subject is currently under study by a collaborative group involving M. D. Anderson, the Institut Gustave Roussy, and the French Genito-Urinary Cancer Group. Results are expected soon. If the independent prognostic role of a rapid decrease in tumour marker levels is established, this might provide a useful tool to better select patients for dose-dense regimens in the future.

The results of the BOP-CISCA-POMB-ACE regimen appear very promising in patients with poor-prognosis NSGCT according to the IGCCCG. However, it is noteworthy that no significant progress has been documented in these patients in phase III trials since 1987. Some investigators believe that a therapeutic plateau has been reached (Nichols *et al*, 1998). Most phase III studies performed in recent years have tested the addition of one concept (e.g. increase in peak dose, use of high-dose chemotherapy plus autologous haematopoietic stem cell support, or the addition of a single new drug) to standard therapies. On the basis of the negative results of these studies, we hypothesise that significant advances may be achieved only by integrating several concepts in a new strategy. This may be achieved by using dose-dense combinations including new drugs that have been recently identified to be active against NSGCT (e.g., paclitaxel, gemcitabine and oxaliplatin) and better identifying patients who are not likely to be cured with the BEP regimen.
